# Developing brain under renewed attack: viral infection during pregnancy

**DOI:** 10.3389/fnins.2023.1119943

**Published:** 2023-08-28

**Authors:** Hatice Recaioglu, Sharon M. Kolk

**Affiliations:** Faculty of Science, Donders Institute for Brain, Cognition and Behavior, Radboud University, Nijmegen, Netherlands

**Keywords:** SARS-COV-2, CHIKV, ZIKV, vertical transmission, pregnancy, brain development, brain barrier, brain inflammation

## Abstract

Living in a globalized world, viral infections such as CHIKV, SARS-COV-2, and ZIKV have become inevitable to also infect the most vulnerable groups in our society. That poses a danger to these populations including pregnant women since the developing brain is sensitive to maternal stressors including viral infections. Upon maternal infection, the viruses can gain access to the fetus via the maternofetal barrier and even to the fetal brain during which factors such as viral receptor expression, time of infection, and the balance between antiviral immune responses and pro-viral mechanisms contribute to mother-to-fetus transmission and fetal infection. Both the direct pro-viral mechanisms and the resulting dysregulated immune response can cause multi-level impairment in the maternofetal and brain barriers and the developing brain itself leading to dysfunction or even loss of several cell populations. Thus, maternal viral infections can disturb brain development and even predispose to neurodevelopmental disorders. In this review, we discuss the potential contribution of maternal viral infections of three relevant relative recent players in the field: Zika, Chikungunya, and Severe Acute Respiratory Syndrome Coronavirus-2, to the impairment of brain development throughout the entire route.

## 1. Introduction

A sudden rise of a viral infection among populations can take a toll on societies by influencing daily life, economy, and public health as it continues to spread. In order to intervene and decelerate the spread as soon as possible, health organizations such as the World Health Organization (WHO) and the National Institutes of Health (NIH) promote research and development on viral pathogens that have the potential to cause widespread health issues (NIH, 2023; World Health Organization, 2023). Nonetheless, extensive spread of viral pathogens is inevitable, especially considering the fast-adapting nature of viruses, climate change, and expanded (inter)national travel (Abdul-Ghani et al., 2020; Pergolizzi et al., 2021). In this review, due to the (i) consistently reported high Zika virus (ZIKV) and Chikungunya virus (CHIKV) case numbers by numerous countries in the recent past, (ii) recent Severe Acute Respiratory Syndrome Coronavirus-2 (SARS-COV-2) pandemic, and (iii) known or potential mother-to-fetus transmission and subsequent neurodevelopmental impact, we have particularly focused on ZIKV, CHIKV and SARS-COV-2 within the context of exposure during brain development. Nevertheless, we realize that TORCH infections as well as HIV infections can still pose a threat to the developing brain in similar ways (NIH, 2023; World Health Organization, 2023); we focus on the “renewed” attack of three of the most recent viral players in the field.

Historically, CHIKV and ZIKV viruses were identified in Africa between the 1940s and 1950s. With recurring outbreaks, CHIKV infections spread through the Indian ocean and Asian regions (2004–2007) reaching the Americas and subsequently leading to the 2013 Caribbean epidemic (Mugabe et al., 2018; Périssé et al., 2020). Travel-associated cases also contributed to its emergence in Europe, for example in Italy (Pierelli et al., 2018). Similarly, ZIKV drew the attention with outbreaks in Higuera and Ramírez (2019) and French Ginige et al. (2021) where 73% and 66% of the population were affected, respectively (Pergolizzi et al., 2021). Growing spread through the Pacific Islands and the Americas as well as reported striking negative pregnancy outcomes were followed by the declaration of ZIKV as an international health emergency by WHO in 2016. In the past 6 years, CHIKV and ZIKV infections have been consistently reported to health organizations reaching 115 and 87 countries, respectively, infecting thousands of people, especially in the Americas region. SARS-COV-2, on the other hand, emerged in Wuhan, China, causing a national outbreak in 2019. Not long after, it was declared as a global pandemic by WHO on 11 March 2020, and is the most recent example of the multifaceted devastating impacts of (global) viral spread. Within months of the emergence of SARS-COV-2, the virus has spread throughout the world, infected millions of people, and caused the death of thousands of people worldwide. These three viruses are still closely monitored by NIH (2023) and World Health Organization (2023).

Extensive spread of viral pathogens raises concern, particularly for pregnant women. Epidemiologic studies of previous pandemics and epidemics have shown that pregnant women and/or their offspring had higher rates of severe illness, morbidity, and mortality (Jamieson et al., 2009; Louie et al., 2010; Charlier et al., 2017; Van Campen et al., 2020; De St Maurice et al., 2021; Ginige et al., 2021). Though overall ZIKV, CHIKV, and SARS-COV-2 do not pose a major threat to pregnant women, upon SARS-COV-2 infection, pregnant women are more likely to require critical care and to have pregnancy complications (Musso et al., 2019; Pomar et al., 2019; Allotey et al., 2020; Schwartz and Morotti, 2020; Jacques et al., 2021; Jafari et al., 2021) and these may have indirect consequences for brain development (Vohr et al., 2017). In ZIKV and CHIKV infections, the main subject of concern had become the neonates due to the observed neurologic and neurodevelopmental abnormalities as a result of vertical transmission (CHIKV, ≥15.5%; ZIKV, 10.9%; Pomar et al., 2017; Contopoulos-Ioannidis et al., 2018; McEntire et al., 2021). Severe neonatal CHIKV cases involving central nervous system (CNS) manifestation was first time reported during Reunion Island outbreak in 2005 (Enserink, 2006; Josseran et al., 2006). Later, it was found that vertically transmitted neonates did not only develop encephalopathy, but also microcephaly and neurodevelopmental delay (Josseran et al., 2006; Borgherini et al., 2007; Couderc and Lecuit, 2009; Gérardin et al., 2014; Ramos et al., 2018; Waechter et al., 2020; Shukla et al., 2021). ZIKV causes congenital malformations which was first suspected during the French Polynesia outbreak but the association could be only made after its arrival to Brazil (Duffy et al., 2009; Hennessey et al., 2016; Mlakar et al., 2016; Moore et al., 2017). Prenatal ZIKV infection can result in a wide range of neurodevelopmental problems including microcephaly, cortical and cerebellar developmental impairment (Mlakar et al., 2016; Yoon et al., 2017; Yuan et al., 2017; Freitas et al., 2020). Despite ongoing debates, it has been generally accepted that SARS-COV-2 can be vertically transmitted during pregnancy (5.3%) and can result in neurological manifestation upon birth (Vivanti et al., 2020; Shook et al., 2022; Vivanti et al., 2022). Although longitudinal cohort studies are still in infancy due to the recent occurrence of the pandemic, emerging data have been pointing out that maternal SARS-COV-2 infection during pregnancy could affect neurodevelopment negatively and even increase the chance of neurodevelopmental and neurologic diagnosis later on (Chevalier and Poillon, 2022; Germano et al., 2022; Shook et al., 2022; Taquet et al., 2022; Wang et al., 2022).

Early brain development spanning from prenatal to postnatal is an intricate and sensitive period. The necessity of rapid but timely and precise changes in the developing brain as well as the relatively naive immune state of the neonates, make the developing brain susceptible to environmental insults including maternal viral infections (Vohr et al., 2017; Elgueta et al., 2022; Jash and Sharma, 2022). Throughout pregnancy, mother and the maternofetal barrier undergo a series of structural and immunological alterations to provide protection against pathogens and ensure healthy development of the fetus until term (Silasi et al., 2015; Cornish et al., 2020). Nonetheless, some viruses have the ability to directly circumvent protective mechanisms and/or induce inflammatory response which can create multi-level alterations. Maternal viral infections can directly and/or indirectly interfere with neurodevelopmental processes and increase the risk for brain injury and brain disorders including neurodevelopmental (NDDs) and neuropsychiatric disorders (NPDs) as well as neurodegenerative diseases (Tomonaga, 2004; Silasi et al., 2015; Zimmer et al., 2021; Elgueta et al., 2022; Ayesa-Arriola et al., 2023). In line with that, in 6% of the prenatally ZIKV-exposed neonates (Martins et al., 2021) and in 51% of the perinatally CHIKV-exposed neonates50 CNS-associated problems were reported, while the proportion of neurological manifestation among neonatal SARS-COV-2 infections was 18% (Raschetti et al., 2020).

Considering recurring outbreaks affecting multiple countries worldwide, and the danger to the developing brain with long-lasting effects, it is important to understand; (I) how viruses can bypass protective mechanisms and barriers, (II) how infections alter the developing brain, (III) how hosts (in this context, both mother and fetus) react to the infection, and (IV) how these reflect onto the developing brain such that it deviates from its developmental trajectory eventually disturbing normal functioning. In this review, by focusing on these points as well as the factors influencing susceptibility of the developing brain to viral infection, our aim is to provide stepwise insights into the effects of viral infection on protective barriers and the developing brain and highlight gaps in the current knowledge which could be helpful in future research of environmental insult-associated impairment of brain development.

### 1.1. Viruses, the maternofetal barrier and brain development

**Figure 1:**
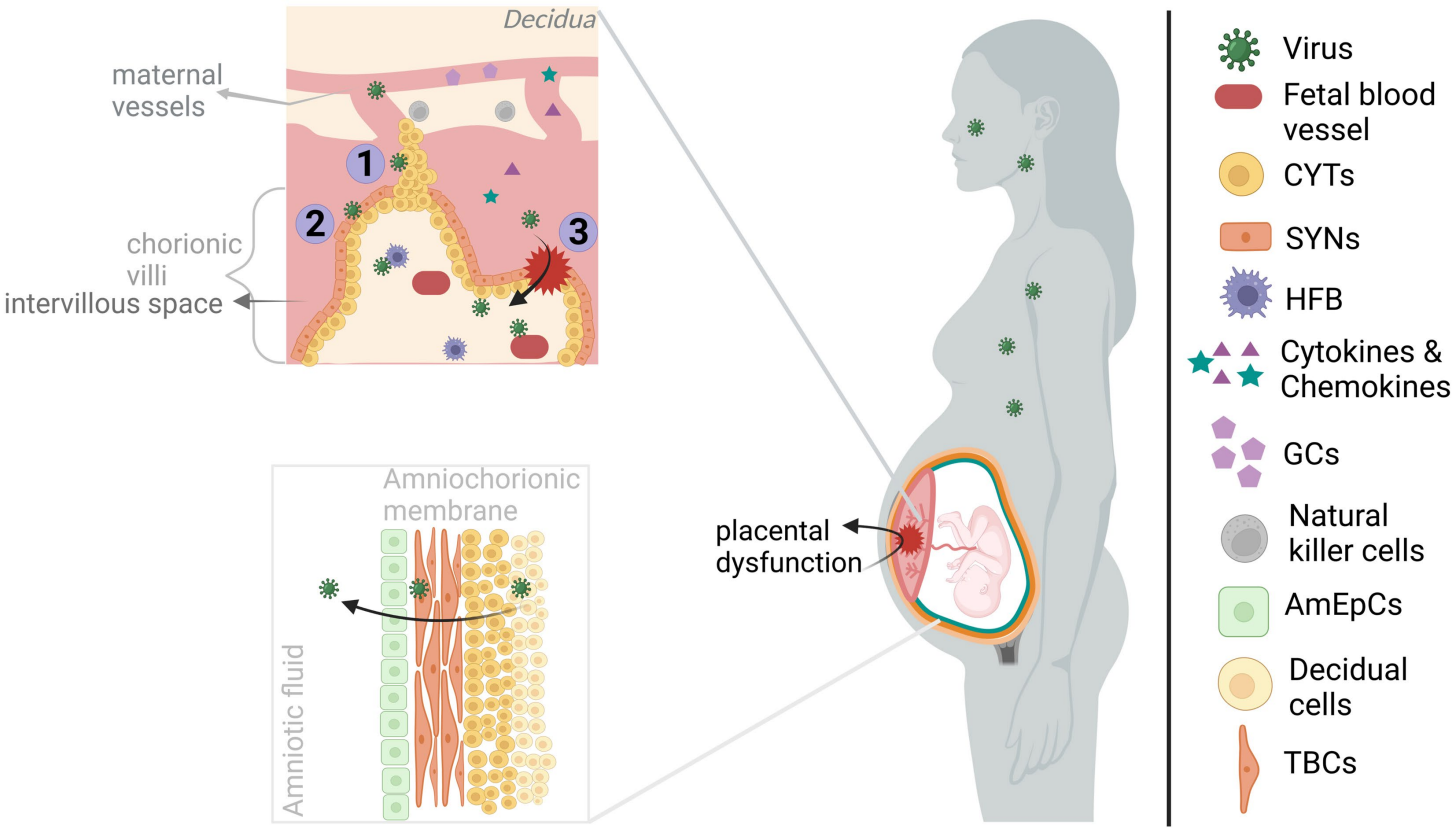
Viral infection of maternofetal barriers. The viruses infecting the mother during pregnancy could reach the fetus by acting on the maternofetal barriers. The maternal side of the placenta, consisting of basal decidua and maternal blood vessels, is in communication with the fetal side of the placenta, containing chorionic villi, mainly through maternal blood filling the intervillous space. However, maternal blood flow is blocked by EVTBs (trophoblastic plugs) during the 1st trimester, and it gradually disintegrates by the beginning of the 2nd trimester. Transplacental transmission of viral infection could occur through (I) infection of/diffusion through the plugs, (II) infection of/transportation across the trophoblasts of chorionic villous, and/or (III) through the structural alteration of the placenta (e.g., via inflammatory response). Upon entering the chorionic villi, viruses can gain access to fetal blood vessels and even directly the developing brain by infecting the HFBs. On the other hand, viruses reaching the amniochorionic membrane are able to elicit the release of viruses into the amniotic fluid, hence, paraplacental transmission. The viruses can directly alter the functioning of the placenta. Also, the maternal and placental inflammatory response might affect placental functioning in addition to allowing the transmission of inflammatory molecules (cytokines and chemokines) to the fetal side. Both cases will have harmful effects on the developing brain. Created with BioRender.com.

The maternofetal barrier with its two main components, placenta and the amniochorionic membrane, develop throughout gestation and create a multicellular complex structure to ensure healthy fetal development by allowing molecule transmission between mother and fetus (e.g., oxygen, nutrients, growth factors) and by providing fetal protection both structurally and immunologically (Figure 1; Pereira, 2018; Silini et al., 2020; Megli and Coyne, 2022). Vertical transmission, the passage of virus from mother to fetus, can occur transplacentally and/or paraplacentally through direct infection of maternofetal cell layers, cell-mediated transport and breach/diffusion.

From a closer perspective, chorionic villi are covered with trophoblast cells (e.g., cytotrophoblasts (CYT) and syncytiotrophoblasts (SYN)) located on the fetal side of the placenta and contain fetal blood vessels as well as fetal macrophages (Hofbauer cells; HFB). CYT and SYN which are in contact with the maternal blood filling the intervillous space, allowing nutrient and oxygen exchange (Megli and Coyne, 2022). Fundamentally, infection of these cells by CHIKV (Gérardin et al., 2014), ZIKV (Tabata et al., 2016; Pereira, 2018; Zanluca et al., 2018; Megli and Coyne, 2022), or SARS-COV-2 (Facchetti et al., 2020; Vivanti et al., 2020) indicates a transplacental viral passage. Especially, infection of the HFB cells by the viruses could be a direct threat to the developing brain due to their migrational ability. That way, they may mediate cell-associated transport into the brain (Tabata et al., 2016; Facchetti et al., 2020; Megli and Coyne, 2022; Vivanti et al., 2022) (see also section 1.3). ZIKV may also diffuse through trophoblastic plugs during the 1st trimester given the susceptibility of extravillous trophoblasts (EVTBs) (Adibi et al., 2016a,b). In addition, viral transportation across barriers can enable transmission. Even in the absence of infection of all cell types, virus-induced cytopathy and a strong immune response could disturb the placental architecture and/or functioning allowing viral transmission, resulting from ZIKV (Miner et al., 2016; Matusali et al., 2019; Cribiu et al., 2021; Ferreira et al., 2021), SARS-COV-2 (Vivanti et al., 2020; Cribiu et al., 2021; DeGrace et al., 2022) and possibly CHIKV (Ferreira et al., 2021) infections. Interestingly, in the earlier CHIKV studies, the absence of placental infection and the presence of perinatal maternal viremia led to the placental breach hypothesis. According to this hypothesis, CHIKV rather than infecting the maternofetal barrier, it infiltrates through the placental breaches during labor when maternal-fetal blood contact occurs (Gérardin et al., 2014; Matusali et al., 2019). The route and gestational time of vertical transmission have importance for the assessment of both preventative options and gestational time-dependent risk for brain development. Although SARS-COV-2 vertical transmission is a rare event, there is clear clustering of reported cases around the 3rd trimester-to-early postnatal period which may be reflective of entry receptor expression-dependent vulnerable period or reporting bias (Allotey et al., 2020; Facchetti et al., 2020; Fenizia et al., 2020; Hosier et al., 2020; Vivanti et al., 2020). Also, sparse CHIKV vertical transmission studies concluded contrasting findings (Gérardin et al., 2014; Contopoulos-Ioannidis et al., 2018; Honorio et al., 2019; Ferreira et al., 2021); hence, further research is required for both viruses.

Amniochorionic membrane attached to the uterine wall (decidua) encapsulates the fetus, thus, enhances fetal protection. The multi-layered membrane is formed with the alignment of the amniotic epithelial cells (AmEpCs), trophoblast cells (TBCs), CYT and decidual cells from most interior (fetal side) to outer surface (maternal side) (Silasi et al., 2015; Silini et al., 2020). Susceptibility of AmEpCs and TBPCs to ZIKV suggests ZIKV diffusion into the amniotic fluid where it may infect fetal skin and/or placenta (Adibi et al., 2016a; Tabata et al., 2016). Similarly, detection of CHIKV in the endometrial epithelium, amniotic fluid, and AmEpCs (Tabata et al., 2016; Platt et al., 2018) and SARS-COV-2 in fetal membranes and amniotic fluid may suggest paraplacental transmission (Fenizia et al., 2020; Penfield et al., 2020; Cribiu et al., 2021).

It should be noted that maternal viral infections during pregnancy is a risk for brain development due to both vertical transmission and maternofetal barrier dysregulation (Yoon et al., 2017; Baines et al., 2020; Shukla et al., 2021; Ayesa-Arriola et al., 2023). As mentioned above, dysregulation can occur via virus-induced cytopathy and inflammatory response. For example, placental cell death and inflammation was reported among ZIKV-infected offspring with neurodevelopmental abnormality (Adibi et al., 2016a,b) and among SARS-COV-2-infected/exposed offspring some of which had a neurological manifestation (Fenizia et al., 2020; Vivanti et al., 2020; Favre et al., 2021). The contribution of placental dysfunction to neurodevelopmental outcome is partially due to its impaired secretory (e.g., neurotrophic factors, serotonin and glucocorticoids) function (Racicot and Mor, 2017; Narang et al., 2021; Megli and Coyne, 2022). Moreover, inflammatory response in the maternofetal barrier can not only cause placental dysregulation, but also affect neurodevelopmental processes in the fetal brain locally (see section 1.5). For instance, prematurity and chorioamnionitis both of which indicating placental dysfunction (Racicot and Mor, 2017; Narang et al., 2021) and were reported in maternal ZIKV (Garcia-Flores et al., 2022; Gomez-Lopez et al., 2022) and SARS-COV-2 infections (Jafari et al., 2021; Wong et al., 2021) could increase the risk for neurodevelopmental disorders (Allotey et al., 2020; Elgueta et al., 2022).

### 1.2. Susceptibility of the developing brain to viral infections

Gestational time of infection, viral tropism, exposed viral load, in combination with the balance between antiviral host immune response and pro-viral strategies are among the factors influencing susceptibility of developing brain to infection. Viral recognition of its entry mediators on host cells, with subsequent viral uptake via endocytosis initiates cellular infection (Agrelli et al., 2019; V’Kovski et al., 2021). The location of the mediators is important since they constitute the target of the viruses. In this way, their localization in maternofetal and brain barriers (e.g., ZIKV: TIM-1, AXL; CHIKV: DC-SIGN, MXRA8, TSPAN9; SARS-COV-2: ACE2) enables vertical transmission and access to the brain (Schnierle, 2019; Feng et al., 2020; Pellegrini et al., 2020; Teixeira et al., 2020; Varma et al., 2020; Xie et al., 2020). The expression pattern of the mediators creates cell-specific tropism of viruses and the distribution of the susceptible cells across the gestational period contributes to gestational time point-associated vulnerability. CHIKV, ZIKV and SARS-COV-2 show overlap in susceptible CNS cell populations, with different cell preference toward astrocytes, neural progenitor stem cell (NPSCs), and mature neurons, respectively (Retallack et al., 2016; Tang et al., 2016; Matusali et al., 2019; Pellegrini et al., 2020; Varma et al., 2020; Yi et al., 2020). It should be noted that none of the CHIKV entry mediators have been specifically associated with brain infectivity. But, the expression pattern of the mediators (e.g., PHB, AXL, FUZ, TIM-1, TSPAN9) some of which are common with ZIKV (Haddad-Tovolli et al., 2017; Garcez et al., 2018; Ramani et al., 2020; Kumari et al., 2021) and the brain injury pattern suggest that CHIKV can infect the brain (e.g., neurons, glial cells, neural stem cells; NSCs) (Das et al., 2015; Racicot and Mor, 2017; Schnierle, 2019; Baines et al., 2020). Interestingly, occurrence of viral infection in the absence of the mediators, especially for CHIKV (Schnierle, 2019; Kril et al., 2021) and ZIKV (Retallack et al., 2016; Hastings et al., 2017) implies employed other routes, presence of unidentified entry mediators and/or interchangeable use of mediators having multiple functions.

Viral infection initiates an antiviral host immune response through viral recognition by pattern recognition receptors (PRRs) such as RIG-I-like receptors (RLRs) and Toll-like receptors (TLRs) leading to release of interferons, inflammatory cytokines, and chemokines (Silasi et al., 2015; Racicot and Mor, 2017; Pereira, 2018). A sufficient level of antiviral response both on maternofetal barrier and in offspring is crucial for viral clearance and to prevent negative outcome. As an example, placental Type-3 interferon (IFN) response (IFN-λ) during the 3rd trimester can prevent ZIKV vertical transmission (Baines et al., 2020). During pregnancy, both maternofetal barrier and fetus have a tolerogenic immune state to prevent fetal rejection. The naïve immune state of the offspring extending to the neonatal period create immature immune responses (e.g., lower innate immune effector, lower IFN response), especially upon viral infection. Together, these create susceptibility to early life viral infections (Kollmann et al., 2017; Cornish et al., 2020). Indeed, insufficient antiviral type-1 IFN has been implicated in neonatal brain infection, developmental delay and severe vertical transmission cases of CHIKV as well as in vertical transmission and brain infection of ZIKV (Miner and Diamond, 2016; Van den Pol et al., 2017). Furthermore, the ability of ZIKV (Adibi et al., 2016a; Adams Waldorf et al., 2018; Lee et al., 2018), CHIKV (Priya et al., 2014; Kril et al., 2021), and SARS-COV-2 (Pellegrini et al., 2020; Song et al., 2021) to evade and/or inhibit Type-1,-2, and/or − 3 IFN responses can contribute vertical transmission, BBB breakdown and/or CNS infection. For instance, SARS-COV-2, inducing metabolic changes in infected and neighboring neurons of cerebral organoids were accompanied with lack of IFN response implying potential contribution of immune response interference in SARS-COV-2 neuropathogenesis (Pellegrini et al., 2020; Song et al., 2021).

To establish successful infection, initial high viral load may not be as crucial for highly neurotrophic viruses like ZIKV (Halai et al., 2017; Adams Waldorf et al., 2018). On the other hand, high dose of exposure to the viruses with lower neurotropism (e.g., CHIKV, SARS-COV-2) together with impaired host immune response might increase the risk for brain infection (Vivanti et al., 2020; Wong et al., 2021). Nonetheless, dose-dependent CNS infectivity of SARS-COV-2 was not consistently reported (Ramani et al., 2020; Yi et al., 2020; Kumari et al., 2021), maybe suggesting a more prominent role of entry mediator expression level compared to viral load.

All three viruses could affect the fetus at any time during pregnancy, especially considering their ability to disturb placental (see section 1.1) and brain barrier (see section 1.3) homeostasis, and to interfere with the development through inflammatory factors (see section 1.5). However, within the context of viral tropism and (anti/pro-viral) immune responses, ZIKV has higher likelihood of affecting the brain during early pregnancy (Miner et al., 2016; Mittal et al., 2022), while CHIKV (Ramos et al., 2018; Waechter et al., 2020), and SARS-COV-2 are more likely to most harmful during late pregnancy (Pellegrini et al., 2020; Vivanti et al., 2020). In line with that, early neurodevelopmental processes (e.g., neurogenesis, migration) are more likely to be affected by ZIKV and late neurodevelopmental processes (e.g., neural circuitry formation and maturation) by CHIKV and SARS-COV-2.

### 1.3. Routes to developing brain and impact of viral infection

**Figure 2:**
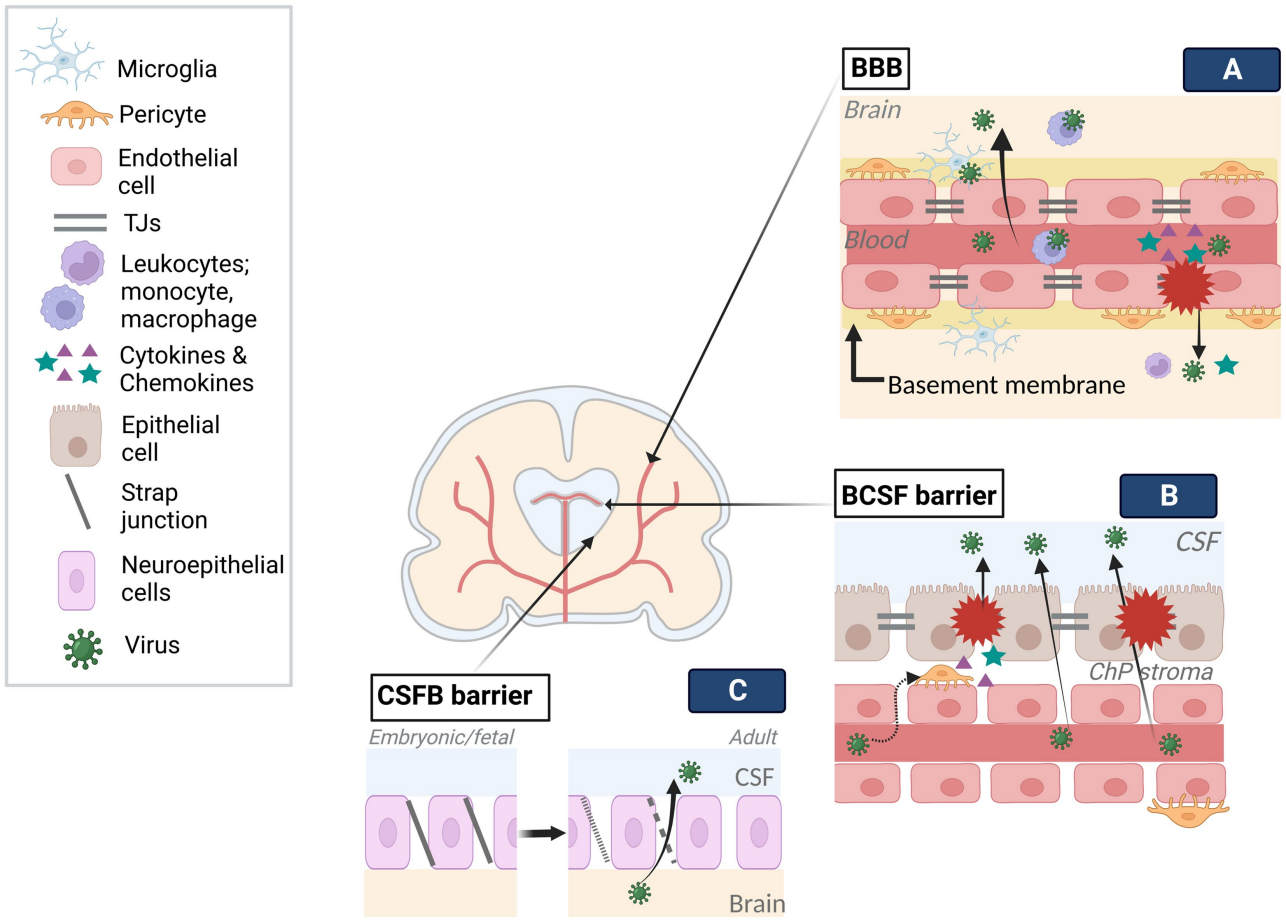
Viral infection of brain barriers. Brain barriers stand as obstacles in the way of viral access to the developing brain. To tackle the limited transmission across barriers, viruses employ various mechanisms. **(A)** Following viral entry into fetal blood circulation, viruses can travel into the blood lumen of the BBB. Infection of endothelial cells can result in viral release into the brain through distinct transcytosis and endocytosis-exocytosis mechanisms. Similarly, the use of immune cells (e.g., leukocytes, microglia) as Trojan horses, can enable viral transmission across the BBB without damaging the barrier. On the other hand, direct modulation of BBB components (e.g., TJs, endothelium) by the viruses and resulting inflammatory response on the barrier can give access to the brain as a result of interrupted barrier integrity. **(B)** Contrary to the BBB, viruses can traffic across fenestrated capillary of the BCSF and reach the ChP stroma. The infection of cells in the stroma, such as pericytes, and/or epithelium could disturb barrier integrity by direct modulation of BCSF components (e.g., TJs, epithelium) or inflammatory response. Trojan horse mechanisms may also be employed in BCSF transmission. **(C)** The strap junctions between neuroepithelial cells, specific to embryonic and fetal stages, form the first brain barrier, limiting the molecule transmission between CSF and the brain. Gradually disappearing strap junctions are replaced by gap junctions of ependyma throughout a period extending to the postnatal stage. The transition from strap to gap junctions may enable viral trafficking. Created with BioRender.com.

In order to affect the developing brain *in utero*, once the virus crosses maternofetal barrier, it needs to cross brain barriers to be able to infect neural tissue (Figure 2). Brain barriers start to form at very early stages, show early functionality, and continue to develop and mature after the postnatal period. Naturally, these structures are different than mature brain barriers: for example, the developing Blood–Brain Barrier (BBB) allows more restricted passage than the mature BBB (Obermeier et al., 2013; Haddad-Tovolli et al., 2017). The embryonic Blood-Cerebrospinal Fluid Brain barrier (e/BCSFB) differently structured than the adult BCSFB, transiently functions during embryonic and fetal stages. Together, the developing BBB and e/BCSFB provide protection from toxins and pathogens, and allow proper development of the brain by creating a controlled internal environment and by adjusting a molecule gradient specific to each developmental time point (Saunders et al., 2018, 2019). Nonetheless, they can be targeted by viruses from the mother during pre/perinatal period which access the developing brain. During that process, the viruses can adopt various strategies which can be generally categorized as with or without barrier disruption.

#### 1.3.1. Blood–brain barrier

Endothelial cells, which are joined together via tight junctions (TJs) restricting paracellular permeability, as well as the basal membrane, which is in contact with pericytes, microglia, and astroglia end feet, constitute the two main components of cerebral blood vessels (Obermeier et al., 2013). By encircling the vessels, they create a highly selective adult BBB. During development, with the appearance of TJs and transporters at gestational week (GW) 12 and becoming more adult-like by GW18, it creates a barrier that pathogens need to cross (Obermeier et al., 2013; Goasdoue et al., 2017; Saunders et al., 2018, 2019). A growing body of evidence indicates that ZIKV can enter the brain mainly without overtly disturbing the BBB permeability via transcytosis (e.g., caveola-dependent transcytosis), endocytosis-exocytosis-dependent replication, and transinfection followed by basolateral release (Tomonaga, 2004; Papa et al., 2017; Leda et al., 2019; Chiu et al., 2020). Similarly, SARS-COV-2 crossing the endothelium via transcytosis (e.g., adsorptive) can breakdown the basement membrane by MMP9-mediated collagen degradation and taken up by several brain regions in an ACE2-dependent manner, which appears to be the main route in SARS-COV-2 encephalopathy cases (Leda et al., 2019; Song et al., 2021; V’Kovski et al., 2021). Limited studies are available showing that the effect of CHIKV on the BBB provides controversial findings: while in murine brain, the BBB was not affected, in zebra fish larvae, both brain vascular endothelium and parenchyma infection without BBB disruption were reported (Couderc et al., 2008; Passoni et al., 2017). However, the mechanism through which CHIKV crossed the BBB could not be assessed. The difference between these findings could be a result of different experimental paradigms (e.g., viral titer, model organism), limited brain endothelial infectivity of CHIKV, and/or low viral release into parenchyma.

The viruses can directly (e.g., cytopathy, interference with developmental processes) and indirectly (e.g., via the immune response) damage the BBB, enabling dissemination into the CNS. For example, (de)phosphorylation-dependent TJ modulation and endothelial cytotoxicity leading to cell death can affect endothelial permeability (Papa et al., 2017; Leda et al., 2019; Buzhdygan et al., 2020; Song et al., 2021). Neurovasculature development starting around GW8, sets the onset of BBB formation and it proceeds in parallel with neurogenesis and brain expansion during which it provides necessary oxygen and nutrients to the cells. Therefore, it is important to have a parallel development of the brain and a proper functioning of the BBB. As such, ZIKV-induced cerebral vasculature developmental delay associated with the reduced neurogenesis, indicated that it affected BBB function (Papa et al., 2017; Garcez et al., 2018; Leda et al., 2019). Likewise, altered protein levels connected to Rho family-associated pathways in CHIKV-infected neonate mice, could disturb blood vessel permeability (Couderc et al., 2008). Receptor ACE2 expression in cerebral vasculature and vascular injury in SARS-COV-2 infection, probably as a result of direct and immune-mediated effects, also pose as a risk factor for the developing brain (Leda et al., 2019; Buzhdygan et al., 2020; Wenzel et al., 2021).

Maternal and placental cytokines produced as an immune response to infection can have detrimental consequences for both the developing brain (see section 1.5) and the BBB. Initiated inflammatory responses against the viral attack, whether it is systemic or local, could disturb BBB permeability through its components (e.g., endothelium, astrocytes, transporters, TJs) resulting in viral entry into the CNS and exacerbation of brain injury. More specifically, activated endothelium upregulates adhesion molecules (e.g., ICAM-1) and inflammatory cytokines (e.g., CCL5, CXCL10, IL-1B, IL-6), allowing recruitment and docking of leukocytes to the BBB as well as increasing permeability (Adams Waldorf et al., 2018; Baines et al., 2020; Fenizia et al., 2020; DeGrace et al., 2022). Passage of leukocytes and viruses into the parenchyma further amplifies inflammatory mediators and BBB breach. Observation of such alterations along with increased matrix metalloproteinases and BBB permeability upon SARS-COV-2 infection indicates inflammation-associated BBB disturbance (Buzhdygan et al., 2020). Similar findings, albeit slight perturbations in TJs and BBB permeability upon ZIKV infection, imply that this may not be the main route of CNS entry, though induced local inflammation followed by subsequent events could amplify brain and barrier damage as well as viral entry (Cle et al., 2020). Indeed, within the brain, dysregulated immune responses associated with vascular damage resulted in leaky BBB and potentially brain calcification (Shao et al., 2016). A dysregulated neuroinflammatory response may also contribute to BBB breakdown in CHIKV infection (Dahm et al., 2016).

Leukocyte (e.g., macrophage, monocyte, microglia) recruitment during or after BBB breakdown confers as a risk factor since they can be hijacked by the viruses for CNS entry with a so-called Trojan horse mechanism (Mustafa et al., 2019). Peripheral monocyte and macrophage infectivity of the viruses further demonstrates their versatility in routes of dissemination and/or persistence (Silasi et al., 2015; Lang et al., 2018; Jafarzadeh et al., 2020; V’Kovski et al., 2021). As an example, increased number of alveolar macrophages with abundant ACE2 expression in severe elderly cases led to the hypothesis of, SARS-COV-2 infection of lungs may enable dissemination to other organs (e.g., brain) via infected macrophages (Abassi et al., 2020; Ferren et al., 2021). Though validity of this mechanism for the fetal stage is not known, in the vertical transmission case, lung infection and brain injury was however reported. Exceptionally, yolk sac-derived microglial cells appearing and migrating to the developing brain (GW4-24; Menassa and Gomez-Nicola, 2018) not only participate in brain development in critical stages but also are the resident macrophages of the brain acting as first-line defenders against pathogens (Tremblay et al., 2020). For example, ablation of microglias, which were localized at the embryonic murine cerebral vessels, decreased not only ZIKV load in brain but also fetal demise (Xu et al., 2020).

#### 1.3.2. Blood-cerebrospinal fluid-brain barriers

Unlike the BBB, inner embryonic CSF (eCSF)-brain and blood-eCSF barriers are the first appearing transient barriers in the developing brain (Goasdoue et al., 2017; Saunders et al., 2019). Both the epithelial blood vessel TJs and the neuroepithelial strap junctions are impermeable to all except smallest lipid soluables as opposed to the adult CSF-brain barrier, hence, create a controlled internal environment and allow expansion of the developing brain until choroid plexus (ChP) becomes functional (Saunders et al., 2018). As these barriers progressively disappear, they become ependyma starting around late 2nd trimester and form the BCSF barrier on the ventricular system (Saunders et al., 2018, 2019). With the initiation of ChP differentiation between GW 6–8, the BCSFB barrier on ChPs forms the 4th, lateral and 3rd ventricles, respectively, until the end of pregnancy (Lun et al., 2015). BCSF barrier on the ChP consists of epithelial cells with TJs on the apical side (Saunders et al., 2018), while the ChP stroma contains endothelial fenestrae with attached pericytes around the blood vessels. ChPs show secretory, barrier, and transportation functions after differentiation, although, similar impermeability pattern as eCSF-brain barrier mentioned above, seem to apply to early differentiated ChPs as well.

Despite restricted molecule transmission between blood-CSF-brain early in development, structural alterations, transitional stages and long-lasting formation of protective ependymal layer may create vulnerability to viral infections (Coletti et al., 2018). As such, it was suggested that BCSF barrier could be vulnerable to ZIKV infection based on its developmental structure, susceptibility of NSCs to ZIKV infection which are closely located to CSF in ventricular zone (VZ) of developing brain, and observed periventricular injury pattern (Nelson et al., 2020). Similarly, CHIKV-infected neonates were claimed to be affected by Trojan horse-associated CNS damage through ChP, leptomeninges, and ependyma due to the subcortical and periventricular damage (Ferreira et al., 2021). Infection on the level of BCSFB barrier can not only cause barrier dysfunction, but also enable viral access to the interior and outer surface of the brain upon viral release into CSF, in both cases there could be negative consequences for brain development. For example, ZIKV-infected pericytes in BCSF barrier disturb ChP epithelial barrier integrity and allow ZIKV CSF entry, likely by releasing factors (e.g., cytokines) (Kim et al., 2020). CHIKV can infect ChP ependymal and leptomeningeal cells and cause severe vacuolization of ChP epithelial cells which could affect its functionality. Productive SARS-COV-2 infection of ChP epithelium initiates cell death and inflammatory responses resulting in functional and structural deficits in the BCSF barrier. Also, decreased production of TTR protein, carrying thyroid hormone from the blood to CSF, may indicate developmental delay if it occurs during gestation (Richardson et al., 2015; Jacob et al., 2020; Pellegrini et al., 2020). Developmental stage-specific CSF volume and component adjustment provides necessary hydrostatic pressure and signaling factors (e.g., differentiation, guidance) to even distant regions. During this process, BCSFB barriers transport the factors from blood to CSF. ChPs play a crucial role by adjusting CSF volume and releasing ChP-derived factors which can affect the behavior of the neural stem cells on the ventricles. Moreover, even at postnatal stage, ChP continues to contribute to the development of the brain such as by modulating cerebral cortex plasticity. Therefore, virus-associated BCSFB damage and viral dissemination into the CSF could be detrimental for the developing brain.

### 1.4. Virus-induced direct damage to developing brain

**Figure 3:**
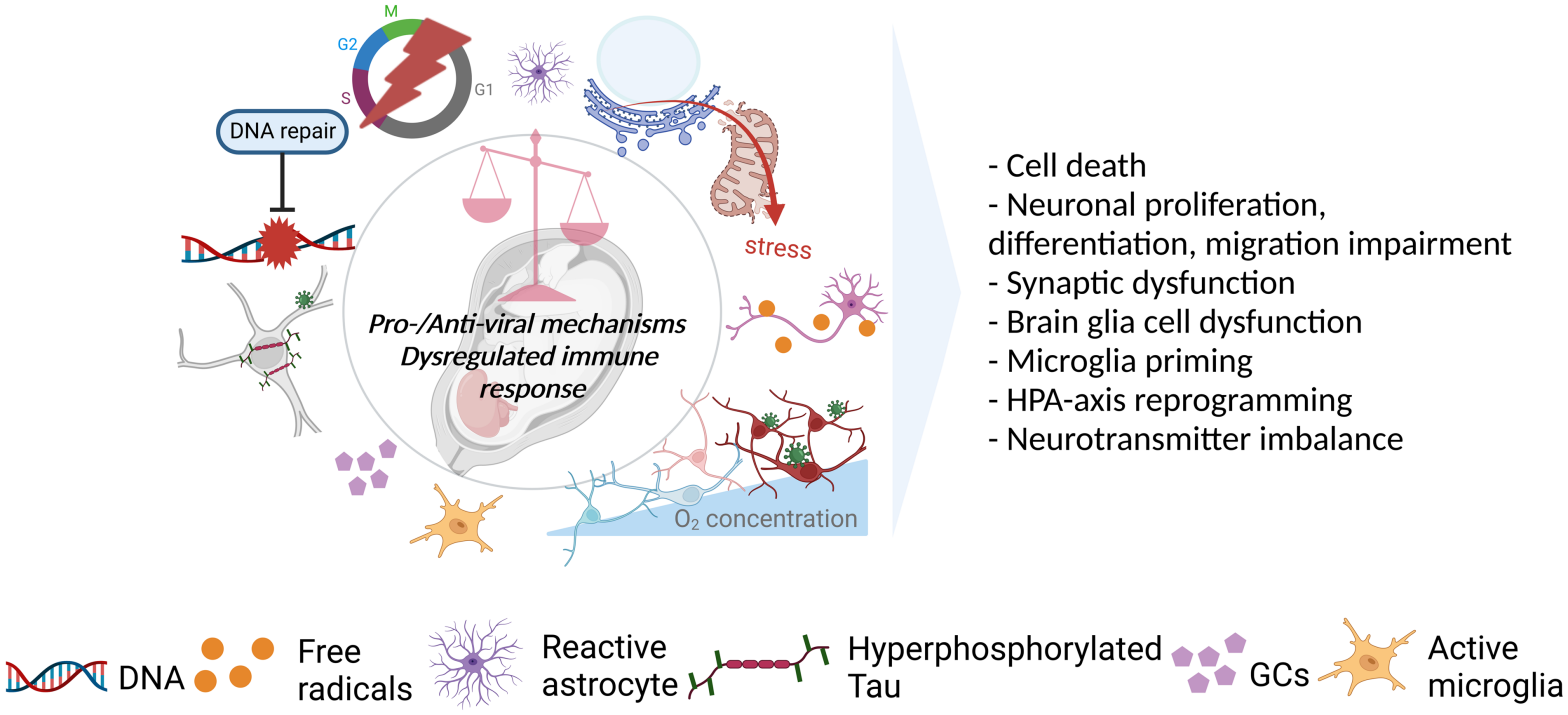
Viral acting mechanisms and consequences for the developing brain. Viral infection initiates a cascade of events in favor (pro-viral) of and against (antiviral) the establishment of persistent, productive infection in the host. The balance between pro-viral and antiviral mechanisms as well as the level and the length of the immune response could dictate the degree to which the developing brain is affected by the infection. As such, the shift in balance toward pro-viral mechanisms coupled with the cytopathic effects (CPEs) of the viruses and strong/prolonged immune response can affect several neurodevelopmental processes in a negative way. As illustrated, viruses can dysregulate several mechanisms including DNA repair, cell cycle, host proteins (e.g., Tau), and cellular metabolism managing the resources (e.g., oxygen), mostly, as a pro-viral strategy. CPEs, including the dysfunction/stress of cellular and sub-cellular structures (e.g., mitochondria, endoplasmic reticulum), in addition to immune response activation leading to, such as, the activation of glial cells (microglia, astrocytes) and release of GCs, can also be observed. These in turn, can have an impact on cell survival, neurodevelopmental processes, neuronal functioning, glial behavior, and neuroendocrine system activity. Created with BioRender.com.

The neurotrophic viruses entering the brain can interfere with the antiviral mechanisms (e.g., apoptosis, autophagy), cellular morphology (e.g., cell lysis, syncytia formation), and functionality (e.g., transcription and translation) through viral replication and/or interaction between viral components and the host. While benefiting from these interferences, such as by enhancing their replication and disseminating within the brain, the viruses create cytotoxicity during the process and temper cellular and molecular events which can damage cell populations and impair proper neurodevelopment (Figure 3). The extent of the impairment depends on several factors such as viral dissemination, targeted cell populations, and developmental time of interference.

#### 1.4.1. Zika virus

ZIKV, having tropism to several cell populations (e.g., glial cells, early-late neurons) in addition to their well-known targets the neural progenitor/stem cells (NP/SCs), can affect the VZ where newborn neurons are generated of different brain regions such as hippocampus, cerebellum, thalamus, and hypothalamus (Cugola et al., 2016; Li et al., 2016; Van den Pol et al., 2017; Shelton et al., 2021). Apart from the presence of entry receptors, axonal transportation, infection through astrocytes and NPC pool, could enhance its dissemination ability (Retallack et al., 2016; Shelton et al., 2021). ZIKV replication and proteins modulating distinct mechanisms such as apoptosis, autophagy, and cell cycle create multi-level impairment in the developing brain spanning from structural defects to (sub−/extra-)cellular alterations (e.g., cytoplasmic vacuolization, mitochondria disruption, axonal rarefaction, and adherens junction impairment) (Miner and Diamond, 2016; Yoon et al., 2017; Lee et al., 2020; Yang et al., 2020). The high impact of ZIKV on neurodevelopment, more specifically on proliferation, differentiation, and migration processes, is due to higher tropism toward NP/SC populations. ZIKV-induced autophagy enhances viral replication by creating favorable conditions for its replication and inhibiting virus-targeted autophagy (virophagy). In the meantime, modulated pathways (e.g., Akt–mTOR, FA) in NSCs, having a dual role in autophagy and brain development, impair neurogenesis (Liang et al., 2016; Tiwari et al., 2020). Commonly observed apoptosis, probably as an antiviral host response, follow an incremental trend over the infection period attenuating brain growth (Cugola et al., 2016; Li et al., 2016). Apoptosis can be initiated in neuronal and glial lineages due to ZIKV-induced transcriptional dysregulation of related genes (Zhang et al., 2016), mitochondrial dysfunction (Yang et al., 2020), oxidative stress (Ledur et al., 2020), and DNA damage (Ledur et al., 2020). Regardless of intrinsic or extrinsic induction of apoptosis, it can be suppressed via stabilization of anti-apoptotic Bcl-2 family proteins by ZIKV (Turpin et al., 2019) demonstrating the extent of self-protective mechanisms especially during early infection while creating a catastrophic environment for the developing brain over time. Furthermore, ZIKV can dysregulate DNA damage repair- and cell cycle-associated (e.g., mitosis, cell cycle process) pathways (Tang et al., 2016; Zhang et al., 2016). As such, directly induced DNA damage combined with cell cycle arrest prevent host DNA replication thereby promoting ZIKV replication (Hammack et al., 2019). ZIKV protein-specific cell cycle arrest at different points with inhibition of differentiation can result in NPC pool depletion (Li et al., 2016; Hammack et al., 2019). During the process, mitotic and centrosomal alterations can interfere with the mode of NPC division (asymmetric/symmetric), chromosome segregation, and cell polarity which can result in chromosomal abnormalities, migration defects, and NPC pool depletion potentially due to chromosomal damage (Gabriel et al., 2017; Kesari et al., 2020).

#### 1.4.2. Chikungunya virus

“The relative less tropism of CHIKV” is in comparison to ZIKV. While the ZIKV can extensively infect neuronal and glial cells, same level of infection was not reported for CHIKV (e.g., cerebral cortex, hippocampus, and cerebellum) during which axonal transportation and syncytia formation play a role (Das et al., 2015; Schnierle, 2019; Ferreira et al., 2021). CHIKV replication with subsequent induction of cell stress, apoptosis, and autophagy mediate its cytopathic effects resulting in cellular damage (e.g., cell lysis, death, cellular disintegration) likely contributing to brain injury (Ramos et al., 2018; Van Ewijk et al., 2021). Although cell type-specific CHIKV vulnerability is not known, higher susceptibility of immature neurons to cytopathy and potential involvement of NS/PCs infection in neurological manifestation was suggested. CHIKV replication and/or interfered cellular processes (e.g., antioxidant enzyme production) can induce endoplasmic reticulum (ER) stress and oxidative stress during which CHIKV can take different routes in modulating the antiviral responses for its benefit. For instance, during ER stress-associated unfolded protein response (UPR) activation, UPR can be suppressed via CHIKV NSP2-mediated host shut-off potentially to evade from UPR-associated antiviral mechanisms (Meshram et al., 2019; Law et al., 2021). The interaction between CHIKV non-structural protein-2 (NSP2) and HSP90-associated PI3K/AKT/mTOR pathway, enables viral replication during early infection. While the occurrence and effects of these interferences in the developing brain is not known, the investigation of the PI3K/AKT/mTOR pathway within the context of CHIKV brain infection may provide information on neurodevelopmental aspects given its importance in neurodevelopment and cell death (Gérardin et al., 2014). Further, independent of stress-induced mTOR inhibition, CHIKV can activate autophagy and apoptosis. Specifically, the interaction of CHIKV NSP2 with human autophagy receptor NDP52, reduces cell death by limiting cell shut off and enhances viral replication by allowing anchorage of the viral replication complex to the Golgi complex (Verlhac et al., 2015). Early induction of autophagy in a glioblastoma cell line and prominent cell death especially in the late stages of the CHIKV brain infection may be the result of skewed autophagy and apoptosis toward a pro-viral role in the CNS (Abraham et al., 2013). Nonetheless, the possibility of host antiviral immune response-associated apoptosis induction cannot be overlooked given simultaneous activation of the immune response (Law et al., 2021). Also, hiding of CHIKV within apoptotic blebs could enable its cell-to-cell spread (e.g., neighboring cells, macrophages), hence, enhance its dissemination. In line with that, bystander apoptosis in murine brain with viral dissemination might be contributed by apoptotic bleb-associated infection (Abraham et al., 2013). Differential expression analyses have revealed modulation of several pathways including synaptic functioning, neurotransmission and neuronal cytoskeletal proteins in addition to cell death and stress response (Lim et al., 2017). However, specific functional connections of these modulations to the observed developmental delay in exposed neonates is not known. Further, despite an observed dysregulated immune response and its known negative influence on neurodevelopment (see section 1.5), a higher likelihood of direct CNS infection in neonates was implied. Therefore, investigation of CHIKV infection in the developing brain by focusing on functional consequences of the directly induced alterations could be informative for directly modulated neurodevelopmental processes.

#### 1.4.3. Severe acute respiratory coronavirus-2

Despite the lower expression of SARS-COV-2 entry-associated proteins in the brain compared to lungs, several brain regions are vulnerable to infection with a preference toward mature neurons (e.g., excitatory, dopaminergic neurons; Jacob et al., 2020; Pellegrini et al., 2020; Lukiw et al., 2022). ZDHHC5, GOLGA7, and ATP1A1 are expressed abundantly during fetal brain development, especially in the 2nd and 3rd trimester, in (im)mature neurons and NPCs unlike widely investigated entry proteins (ACE2 and TMPRSS2; Varma et al., 2020; Chen et al., 2021). While showing the potential danger to the developing brain, the *in vivo* and *in vitro* assessment of newly identified interactors is still lacking. The dissemination within the brain might be contributed by syncytia formation allowing cell-to-cell spread (Jacob et al., 2020; Zhang et al., 2020) and axonal transport given its ability to mimic relevant transport proteins (Yapici-Eser et al., 2021). Cell death, particularly at the proximity of infected cells, has been commonly reported within the context of brain infection which could be contributed by inflammatory response (Jacob et al., 2020; Ferren et al., 2021) and cellular dysfunction (Song et al., 2021; Valeri et al., 2021). For example, neuronal metabolic alterations can manage cellular resources to both un/infected neurons probably for viral replication and lead to death of nearby neurons as a result of hypoxic-state (Song et al., 2021). SARS-COV-2-induced oxidative stress, either as a result of mitochondrial manipulation (Clough et al., 2021) or facilitated infection, leads to DNA damage that cannot be repaired and enhances cortical neuronal death. Further, cell cycle impairment, which could be due to the DNA damage and/or oxidative stress, could induce neuronal senescence in which proliferation is permanently inhibited (Valeri et al., 2021). While SARS-COV-2 does not seem to have teratogenic effects similar to ZIKV, loss of neuronal populations during 2nd–3rd trimester when cortical growth continues may create subtle changes, contributing to cognitive and behavioral alterations (Andescavage et al., 2017). SARS-COV-2 interacting with host proteins could cause their dysfunction and affect relevant processes negatively (Idrees and Kumar, 2021; Yapici-Eser et al., 2021; DeGrace et al., 2022; Hok et al., 2022). For instance, the viral heparin binding site could assist binding of relevant proteins (e.g., Aβ, α-synuclein, tau, prion) and lead to their aggregation and neurodegeneration (Idrees and Kumar, 2021). Similarly, as the hallmark of adult-onset taupaties, mislocalized and aberrantly phosphorylated Tau was reported in cerebral organoids (Ramani et al., 2020, 2021). Given the role of Tau on axonal microtubule organization during neural differentiation as well as synaptogenesis and dendritic spine formation, dysfunctional Tau could have consequences for the developing brain (Rankovic and Zweckstetter, 2019). Additionally, viral interaction with MAO, growth factors, and the proteins having role in synaptic and neurotransmission could misbalance neurotransmitter levels, affect neuronal survival and neuronal differentiation (Yapici-Eser et al., 2021; Hok et al., 2022). Despite recent occurrence of the pandemic, emerging information suggests neurobiological interference of SARS-COV-2 creates a neurotoxic environment, though mechanisms and functional consequences especially for developing brain require further investigation.

### 1.5. Immune activation, inflammatory mediators and developing brain

Maternal viral infection inducing immune response which is mediated by inflammatory factors can change the homeostasis of the barriers and the developing brain, as mentioned in previous sections. During maternal infection, inflammatory cytokines in the fetus can increase due to the transplacental passage, placental production or fetal production posing as a developmental stressor for fetus (Fenizia et al., 2020; Ferreira et al., 2021; Han et al., 2021). Specifically, pathogen/damage-associated molecular patterns recognized by PRRs (e.g., TLRs) which are expressed in the CNS (e.g., neurons, microglia, astrocytes) and peripheral immune cells (e.g., macrophages) mediate cytokine release. Not only this molecular pathway was suggested to be the link between maternal inflammatory factors and immune-mediated disruption of brain development but also cytokines are recognized as the key modulators of developmental trajectories (Han et al., 2021).

Even a slight change in the balance between pro- and anti-inflammatory factors released upon pathogen encounter can be enough to deviate from normal neurodevelopment. Evidence suggests that all three viruses can induce immune activation and cause dysregulated immune response. Firstly, fever, associated with pyrogenic cytokines (IL6, IL1b, and TNFα) is among the symptoms in infected pregnant women and/or exposed neonates (Dahm et al., 2016; Raschetti et al., 2020; Ginige et al., 2021). Secondly, enhanced level of cytokines and chemokines (e.g.IL1B, IL6, CCL5-2, CXCL9-10, and TNFα) in the mother, placenta, and/or neonate was reported in SARS-COV-2 (Fenizia et al., 2020; Cribiu et al., 2021; DeGrace et al., 2022) and ZIKV (Lima et al., 2019; Rabelo et al., 2020) infections. Further, there is an association between SARS-COV-2 and cytokine storm (DeGrace et al., 2022) as well as between maternal cytokines and fetal brain abnormalities in ZIKV infection (Adams Waldorf et al., 2018). In case of CHIKV, maternofetal cytokine transmission is likely given the higher level of cytokine release during the acute phase of the infection compared to the convalescent phase (Kril et al., 2021). Prenatal exposure to some cytokines at high levels which are also seen in the viral infections (e.g., IL6, IL17) is sufficient to drive a behavioral outcome (Venugopalan et al., 2014; Chirathaworn et al., 2020). But maternal immune activation (MIA) is seen as a disease primer due to absence of neonatal neuropathology in most cases (Jiang et al., 2018). Thirdly, neuroinflammation and/or glial activation was demonstrated individually for all viruses mentioned (Dahm et al., 2016; Raschetti et al., 2020; Han et al., 2021; Elgueta et al., 2022). Finally, ZIKV (Cle et al., 2020; Rabelo et al., 2020), CHIKV, and potentially SARS-COV-2 (DeGrace et al., 2022) can cause prolonged immune activation. Several routes have been postulated through which immune activation and inflammatory responses may alter the developing brain, thus, exacerbate brain injury and/or predispose to NDDs, NPDs such as via glial cells, trained immunity, and HPA-axis (Figure 3).

Glial cells namely astrocytes and microglia play an important role during development enabling functional neural circuitry formation, maturation and maintenance (Lago-Baldaia et al., 2020; Eze et al., 2021). In response to infection and cytokine release, glial cells become activated showing a pro-inflammatory state to improve neuroprotection and homeostasis (Dahm et al., 2016; Cornish et al., 2020; Elgueta et al., 2022; Kim et al., 2022). However, such early life stresses can be damaging to the developing brain due to a dysregulated glial functioning (e.g., impaired phagocytic activity, over/prolonged activation) and blunted glial development. Phagocytosis, required for synaptic pruning by astrocytes and primarily by microglia, is one of the functions that is found to be impaired upon MIA and is associated with NDDs (Lago-Baldaia et al., 2020; Carloni et al., 2021). Astrocytes, having a role in synaptogenesis, synapse regulation and neurotransmitter turnover, upon overactivation can release neurotoxic molecules as well as causing excitotoxicity due to impaired neurotransmitter turnover function resulting in neuronal dysfunction and cell death (Inglis et al., 2016; Lago-Baldaia et al., 2020; Linnerbauer et al., 2020; Stasenko et al., 2023). Further, microglia can amplify not only excitotoxic activity of astrocytes but also fetal brain injury via, e.g., secreted cytokines and free radicals (Linnerbauer et al., 2020). For example, the myelinating cells, pre-oligodendrocytes, are vulnerable to cytokines partially due to their inability to scavenge free radicals efficiently. Thus, potential damage can result in hypomyelination and even white matter injury (Motavaf and Piao, 2021; Stasenko et al., 2023). That may contribute to brain injury and developmental abnormality in CHIKV-exposed infants considering oligodendrocyte susceptibility to the infection, and the presence of inflammatory response, demyelination, and white matter injury (Gérardin et al., 2014; Das et al., 2015; Mehta et al., 2018; Ramos et al., 2018). Moreover, microglial activity could contribute disruption of oligodendrocyte development in Zika infections (Li et al., 2016).

Microglias, as the primary immune cells of the CNS, as well as peripheral immune cells (e.g., macrophages, monocytes) are particularly relevant within the context of trained immunity and long-term impact of the prenatal immune activation. Developmental stressors (e.g., infection or cytokine exposure) (1st hit) can induce immune training by epigenetic and metabolic reprogramming immune cells (priming) thus enabling them to create strong inflammatory responses to the subsequent stimulus (2nd hit) (Netea et al., 2020; Carloni et al., 2021). Particularly microglia priming could be the key mediator of the negative consequences (e.g., neuronal and behavioral abnormalities) of the developmental stressor since MIA alters the function of microglia to the subsequent stimulus. Moreover, compared to adult microglias, neonatal microglias are more prone to priming (Carloni et al., 2021). For example, developmental stressor-induced inflammatory response, by priming microglias, created susceptibility to Alzheimer’s disease. As such, late low-dose Aβ treatment exacerbated microglial activation contributing to synapse damage and cognitive impairment (Frost et al., 2019). A potential role of trained immunity in ASD onset and progression was suggested with the observations of altered immune response to the subsequent stimuli along with fluctuating neuropsychiatric symptoms in a subset of the ASD children in the cohort.

Infection and cytokines (e.g., IL-1/2/6, TNFα) by affecting hormone release from Hypothalamus, Pituitary and Adrenal glands activate maternal and/or fetal HPA axis leading to release of glucocorticoids (GCs) as an end product (Han et al., 2021). HPA-axis activity is controlled by a negative feedback loop during which produced GCs inhibit its continuous activation, preventing excess GC exposure. However, cytokines can not only downregulate placental GC inactivating enzyme (Cottrell and Seckl, 2009) but also create GC resistance upon prolonged exposure, thus, exposing the fetus to unrestrained GC. Also, GCs can suppress inflammation through production of anti-inflammatory cytokines (Han et al., 2021). Therefore, the ability of the developing brain to cope with inflammation partially depends on sufficient stress response generation through the HPA-axis. And that may differ before late gestation and during early postnatal period considering the functionality of the HPA axis throughout development (Sheng et al., 2020). Altogether, these can permanently alter HPA-axis response to stress (e.g., hyperactivation) which can contribute behavioral alteration (e.g., anxiety) and vulnerability to several diseases (e.g., psychiatric) in adulthood (Cottrell and Seckl, 2009; Han et al., 2021). Further, HPA-axis’ hyperactivation could affect development of neurotransmitter systems and neurotransmitter levels in the developing brain due to the connection between neurotransmitter systems (e.g., serotonergic, dopaminergic) and the HPA-axis.

## 2. Conclusion and perspectives

Viruses whether it is due to viral receptor expression or placental breach can reach the fetus and move toward the fetal brain. But knowing the route, vulnerable developmental timing and the type of dysfunction can help to better assess the risk for brain development. The viruses inherently trying to establish productive and persistent infection can affect distinct developmental processes such as neuronal proliferation, differentiation as well as synaptic and brain barrier function especially considering the naïve immune state of offspring. Further, virus-induced dysregulated immune responses could have long-lasting effects on the developing brain. Better identification of the targeted cellular processes with respect to brain development for CHIKV and SARS-COV-2, additionally, the effects of dysregulated immune response upon CHIKV, ZIKV, and SARS-COV-2 infection on developing brain can help understanding the scope of neurodevelopmental impact. And that could enable development and/or application of the targeted therapies for the affected newborns.

## Author contributions

HR wrote the manuscript with support from SK. All authors contributed to the article and approved the submitted version.

## Conflict of interest

The authors declare that the research was conducted in the absence of any commercial or financial relationships that could be construed as a potential conflict of interest.

## Publisher’s note

All claims expressed in this article are solely those of the authors and do not necessarily represent those of their affiliated organizations, or those of the publisher, the editors and the reviewers. Any product that may be evaluated in this article, or claim that may be made by its manufacturer, is not guaranteed or endorsed by the publisher.
